# Regulatory mechanisms of neuro inflammation from a gender perspective: interactions among astrocytes, sex hormones, and the gut-brain axis

**DOI:** 10.3389/fnagi.2025.1675694

**Published:** 2025-09-24

**Authors:** Jia-Wei Shi, Xiu-Li Meng, Yu Ye, Yi-Ting Wang, He-Xuan Ding, Wen-Xu Qi, Rui Feng, Ke Zhang, Ming Lei

**Affiliations:** ^1^Department of Developmental Cell Biology, Key Laboratory of Cell Biology, Ministry of Public Health, China Medical University, Shenyang, China; ^2^Department of Environmental Health, School of Public Health, China Medical University, Shenyang, China; ^3^Department of Neurology, The First People' s Hospital of Shenyang, Shenyang, Liaoning, China; ^4^Department of Radiology, Shengjing Hospital of China Medical University, Shenyang, China; ^5^Department of Pharmaceutical Toxicology, School of Pharmacy, China Medical University, Shenyang, China; ^6^Key Laboratory of Medical Electrophysiology, Ministry of Education & Medical Electrophysiological Key Laboratory of Sichuan Province, Institute of Cardiovascular Research, Southwest Medical University, Luzhou, China

**Keywords:** neuro inflammation, gender differences, astrocytes, sex hormones, gut-brain axis, aging

## 1 Introduction

The rapid aging of the global population represents one of the most significant social challenges of the twenty first century, accompanied by a sharp increase in age-related diseases (Kaizu and Miyata, 2025; Reinehr et al., 2024). Among these, the progressive decline of the nervous system plays a central role, impairing mobility, perception, autonomic regulation, and cognition, thereby reducing the quality of life and independence of the elderly (Huang et al., 2025). Substantial research indicates that neuro inflammation is a key mechanism in neurological disorders and cognitive aging (Marino et al., 2022; Wang et al., 2025).

Extensive epidemiological and clinical evidence has demonstrated significant gender differences in the incidence, progression, and treatment responses of many age-related diseases, with females often exhibiting greater vulnerability or distinct clinical manifestations (Migliore et al., 2021). Biological sex is thus a fundamental variable influencing disease susceptibility. Foundational studies confirm that immune responses and neuro inflammatory pathways show sexual dimorphism (Shabab et al., 2017), shaped by sex hormones, sex chromosome genes, and sociocultural factors.

Understanding gender differences in neuro inflammation during aging is crucial for elucidating disparities in neurodegenerative diseases and for developing gender-specific therapeutic strategies. This requires attention not only to molecular mechanisms but also to hormonal dynamics and immune-neural interactions. This article focuses on astrocytes, sex hormones, and the gut-brain axis in modulating gender differences in neuro inflammation, aiming to inform clinical research and targeted therapies (Table 1).

**Table 1:** Gender differences, potential mechanisms and clinical significance in the regulation of neuroinflammation.

**Regulatory factors**	**Gender differences manifestation**	**Potential mechanism**	**Clinical significance / research direction**
Astrocyte	-Male: Low expression of the ERBB4 gene, enrichment of apoptotic pathways -Female: High expression of CCL2 and INHBA genes, active Wnt signaling pathway	-ERBB4 is regulated by estrogen and affects synaptic plasticity. -CCL2 promotes inflammation, while INHBA is involved in the activation of the activin A/TGF-β pathway regulation.	-Male: Neuroprotective drugs targeting the apoptotic pathway -Female: Utilizing the Wnt pathway to enhance anti-inflammatory capabilities
Sex hormone	-Male: Dominated by androgens, with low estrogen levels. -Female (before menopause): High estrogen levels, normal ERBB4 expression, anti-inflammatory	-Estrogen promotes neuroinflammation through the PI3K/Akt pathway.-Estrogen binds to the ERBB4 promoter to regulate gene expression	- Postmenopausal women: Optimization of estrogen replacement therapy - The gender-specific application of sex hormone receptor modulators
Gut-brain axis(BGMA)	-Male: Differences in the composition of gut microbiota, and variations in the metabolism of short-chain fatty acids.-Woman: Diversity of intestinal flora, enrichment of lactobacilli	-Estrogen regulates the microbiota through the “estrobolome” network.-The microbiota affects the hepatic and intestinal circulation of estrogen through β-glucuronidase.	-The gender-specific application of probiotics/ fecal microbiota transplantation-Regulating microglial cell activity by targeting the SCFAs-GPCR pathway
Immune system	-Male: The inflammatory pathways in neurodegenerative diseases are different. -Female: High risk of autoimmune diseases, strong inflammatory response	-Sex hormones (such as estrogen) regulate the activation of microglia cells.-Gender dimorphism of X chromosome immune-related genes	-Gender-based stratified immunomodulatory therapy-Exploring the influence of exploratory chromosome genes on neuroinflammation.
Other factors	-The social gender factor (medical resources, health behaviors) indirectly amplifies the differences in diseases.	The interaction between the environment and biological factors (such as the impact of stress on the gut microbiota)	Interdisciplinary research (social psychology + neuroimmunology)- A longitudinal study on the temporal and spatial dynamic changes in the elderly population

### 2.1 Astrocytes

Astrocytes can be categorized into homeostatic astrocytes and reactive astrocytes, forming an integral component of the innate immunity within the central nervous system (CNS) (Tyurikova, 2024). These cells are capable of producing a variety of cytokines, chemokines, and other immune factor reservoirs (Wiese et al., 2012; Jensen et al., 2013; Farina et al., 2007). Astrocytes engage in complex intercellular communication with neurons, oligodendrocytes, microglia, and other cell types in the CNS, including bidirectional crosstalk with microglia during neuroinflammatory responses (Singh, 2022). The neuroinflammation mediated by astrocytes plays a significant role in neurodegenerative diseases, such as Alzheimer's disease and Parkinson's disease. Notably, there are sex-specific differences in the gene expression profiles of astrocytes involved in the regulation of neuro inflammation.

During the modulation of neuro inflammation, male-specific alterations in astrocytes have been observed, such as a significant downregulation of the ERBB4 gene, which encodes a receptor tyrosine kinase essential for neurodevelopment and synaptic plasticity. Abnormal expression of this gene may trigger synaptic dysfunction and neuroinflammation (Santiago et al., 2022). In contrast, female-specific changes include the upregulation of the CCL2 (C-C motif chemokine ligand 2) gene and the INHBA (inhibin subunit beta A) gene. CCL2, which encodes a monocyte chemoattractant protein-1, serves as a crucial regulatory factor in neuro inflammation (Posillico et al., 2021), exhibiting significantly higher expression levels during neuroinflammatory modulation. INHBA encodes a subunit of activin A, a member of the transforming growth factor beta (TGF-β) superfamily, which is involved in the regulation of neuroinflammation (Wu et al., 2021).

Furthermore, a notable enrichment of apoptosis and programmed cell death processes is present in male astrocytes, contrasting with alterations in Wnt signaling and cell cycle transition pathways observed in female astrocytes. This indicates profound differences between the sexes in their neuroinflammatory responses (Soudy et al., 2025). Investigating these sex-specific cellular mechanisms may enhance our understanding of the role of sex in neurobiology and provide new avenues for personalized medicine. For instance, drug development targeting apoptotic pathways in male astrocytes could potentially improve prognosis in male patients suffering from neurological disorders (Jan and Chaudhry, 2019). Concurrently, leveraging the protective mechanisms associated with female astrocytes, future research could explore how modulation of Wnt signaling pathways might augment cellular anti-inflammatory capacities, thereby offering new therapeutic strategies for the repair of the nervous system.

In addition to astrocytes, other neural cell types also play critical roles in the gender-specific regulation of neuro inflammation. Microglia, the resident immune cells of the CNS, display marked sexual dimorphism in their density, morphology, and activation thresholds (Li et al., 2024). Female microglia tend to mount stronger pro-inflammatory responses, which has been linked to the higher incidence of autoimmune and neuro inflammatory disorders in women, whereas male microglia may exhibit greater resilience to certain inflammatory challenges (Gildawie et al., 2020). Oligodendrocytes and their progenitor cells also contribute to sex differences, as they are essential for myelination and remyelination after injury (Long et al., 2021). Evidence suggests that estrogen and androgens can differentially regulate oligodendrocyte survival and maturation, thereby influencing white matter integrity in a sex-dependent manner (Zahaf et al., 2023).

### 2.2 Sex hormones

Sex hormones are a class of significant chemical substances secreted by endocrine glands, primarily consisting of estrogens, progestogens, and androgens (McEwen and Milner, 2017). These hormones play a crucial role in the regulation of neuro inflammation, particularly estrogen. Intuitively, the marked differences in estrogen and androgen levels between males and females result in gender-specific variations in the regulatory roles of sex hormones in neuro inflammation (Lu et al., 2025). However, the mechanisms through which sex hormones influence neuro inflammation are notably complex and extensive (Amanollahi et al., 2023), including effects on the activation of microglial cells and influences on the gut microbiota, the latter of which will be elaborated upon in the context of the gut-brain axis.

Sex hormones exert regulatory effects on numerous genes associated with neuro inflammatory responses. Taking the previously mentioned astrocytic ERBB4 gene as an example, this gene is regulated by estrogen, with its promoter region containing an estrogen response element half-site overlapping a binding site for activator protein-1 (Zhu et al., 2006). Upon stimulation by estrogen, this regulatory element enhances the binding of estrogen receptors to the intracellular domain of ERBB4 (4ICD), thereby modulating the expression of ERBB4. Given that estrogen levels in males are significantly lower than in females, ERBB4 is markedly underexpressed in males compared to females, subsequently promoting neuroinflammation; conversely, the normal expression of ERBB4 in females serves an anti-inflammatory function. Nevertheless, the decline in estrogen levels in aging females, particularly post-menopause, may increase the risk of neuroinflammation, indirectly corroborating the higher incidence of neuroinflammatory-related diseases (such as neurodegenerative disorders) in females compared to males (Bianco et al., 2023).

A bidirectional regulatory network exists between sex hormones and neuroinflammatory genes. For instance, as previously discussed, INHBA plays a critical role in follicular development and hormonal regulation in the ovaries, while the overexpression of INHBA can decrease the secretion of activin and estradiol while simultaneously increasing the secretion of inhibin and progesterone (Wu et al., 2021). Therefore, the INHBA gene is involved both in the regulatory processes of sex hormones and in mechanisms of neuroinflammation and neuroprotection. This illustrates the complexity of the body's regulatory processes concerning neuroinflammation, highlighting the pivotal interactions among various factors (Wu et al., 2021).

Additionally, estrogen plays an important role in neurogenesis, particularly evident in the dentate gyrus region of the hippocampus, where its neuroprotective effects are mediated through the enhancement of the PI3K/Akt signaling pathway (Harms et al., 2001).

Taken together, current evidence indicates that sex hormones exert dual and context-dependent effects on neuroinflammation. Estrogen is generally regarded as anti-inflammatory and neuroprotective, as seen in Alzheimer's disease and Parkinson's disease, where it reduces excessive microglial activation and supports neuronal survival (Wu et al., 2016). However, under certain pathological conditions, estrogens may also enhance immune activation, thereby exerting pro-inflammatory actions (Dragin et al., 2017). In contrast, androgens are more often associated with immunosuppressive and anti-inflammatory roles, although the supporting data remain less consistent (Gubbels bupp and Jorgensen, 2018). Clinical observations further highlight these dynamics: in multiple sclerosis, which disproportionately affects women, elevated estrogen levels during pregnancy are associated with symptomatic improvement, while disease activity often rebounds postpartum (Voskuhl and Momtazee, 2017); in Alzheimer's disease, the postmenopausal decline in estrogen contributes to the increased female susceptibility (Ali et al., 2023). These findings suggest that sex hormones are not uniformly protective or harmful but rather act in a disease- and stage-specific manner, reinforcing the need for carefully designed, sex-stratified therapeutic strategies.

### 2.3 Gut-brain axis

The diversity of gut microbiota is considered to be associated with gender, which is a significant factor influencing the intestinal microbiome, resulting in disparities in the composition and types of gut microbiota between males and females at birth (Rio et al., 2024). These differences in gut microbial composition also lead to gender-specific variations in responses and susceptibilities to related diseases.

The Brain-Gut Microbiome Axis (BGMA) plays a crucial role in brain-related disorders. Short-chain fatty acids (SCFAs) in the gut activate microglial cells through G protein-coupled receptors (GPCRs) (Kartjito et al., 2023). When the intestinal barrier function is compromised due to dysbiosis, abnormal immune system responses interact with the nervous system, ultimately leading to a state of “neuroinflammation”(Loh et al., 2024).

The immune system plays a pivotal role in the bidirectional communication between the gut microbiota and the brain (Yoo et al., 2020). BGMA regulation by the immune system is a particularly intriguing mechanism contributing to gender-related differences in health risks. Gut microbiota and sex steroids jointly modulate immune function, and further research on specific bacterial metabolites that regulate immunity could inform dietary interventions for preventing or treating autoimmune diseases (Rizzetto et al., 2018). Gender differences in microglial characteristics may also differentially influence sensitivity to pro-inflammatory and anti-inflammatory products of the microbiome (Kodama and Gan, 2019).

Sex hormones influence the brain-gut microbiome axis at multiple levels, including the central nervous system, enteric nervous system, and enteroendocrine cells. Numerous studies have demonstrated the significant role of sex hormones in the formation and regulation of gut microbiota (Kaliannan et al., 2018; Sakamuri et al., 2023). Specifically, the effects of sex hormones are reflected both in the absolute abundance of certain bacterial taxa and in the relative proportions of microbial communities. For example, estrogens selectively increase the quantity of Lactobacillus species, while also reshaping the overall microbial composition by enhancing community diversity in females compared with males (Siddiqui et al., 2022). In addition, sex hormone levels modulate functional microbial outputs such as SCFA production, thereby indirectly influencing inflammatory and neuroimmune pathways (Dalile et al., 2019). Conversely, the microbiome plays a crucial role by modulating sex hormones. Circulating estrogen levels are significantly regulated by the microbiome, and when the microbiome is disrupted, estrogen levels decrease accordingly (Baker et al., 2017). The gut microbiota regulates the enterohepatic circulation of estrogen through β-glucuronidase, forming the “estrobolome” network (Baker et al., 2017; Plottel and Blaser, 2011); estrogen metabolites, in turn, selectively promote the proliferation of specific bacteria (such as Lactobacillus), revealing a positive feedback mechanism (Kwa et al., 2016). Androgens (particularly testosterone) similarly affect the activity of the gut-brain axis, partly promoting the conversion of estradiol (Zuloaga et al., 2020). Thus, it is evident that gut microbiota and sex hormones jointly act on the regulation of neuroinflammation, a process exhibiting gender differences.

During the aging process, significant changes occur in the gut microbiota, accompanied by a decrease in sex hormones. Therefore, it seems worthwhile to further explore how the interactions between sex hormones and gut microbiota, as well as their regulation of neuroinflammation, may change. In clinical treatment, it is noteworthy to consider gender-specific therapeutic effects of gut microbiota interventions (such as probiotics/fecal microbiota transplantation).

## 3 Conclusion

Gender disparities in neuroinflammation highlight the need for precise, gender-specific therapies, particularly in the elderly. Future studies should use organoid models and gender-stratified trials to test interventions targeting astrocytes, sex hormones, and gut microbiota. The immune system remains central, with BGMA–hormone regulation requiring deeper investigation. Social factors, such as healthcare access and behaviors, also amplify disparities.

Spatiotemporal dynamics of neuroinflammation in aging demand longitudinal studies, while interactions among astrocytes, microglia, and peripheral immune cells can now be dissected with single-cell sequencing. Advances in genomics and epigenetics further identify sex-specific genes and networks as therapeutic targets. Despite challenges in translating animal findings and modeling hormone dynamics, interdisciplinary efforts will be key to progress.
